# Scrolling for Change: Using Instagram to Disseminate Evidence-Based Brief Video Interventions to Empower Childhood Trauma Survivors

**DOI:** 10.1192/j.eurpsy.2025.305

**Published:** 2025-08-26

**Authors:** S. Haim-Nachum, D. Amsalem

**Affiliations:** 1 Department of Psychiatry, Columbia University, New York, United States; 2 School of Social Work, Tel Aviv University, Tel Aviv, Israel

## Abstract

**Introduction:**

Childhood trauma (CT), especially experiences of abuse and/or neglect, is highly prevalent. Although CT significantly impacts mental health, those who experience it often avoid seeking treatment. Stigma –negative beliefs surrounding mental-health and trauma, is a primary obstacle to seeking care. We recently developed and demonstrated the efficacy of a brief video intervention designed to reduce stigma and increase openness to mental health care among CT survivors. This intervention was tested in a controlled environment (crowdsourcing platforms) to ensure that broader dissemination would cause no harm. Given the central role social media plays in the lives of youth, it is crucial to explore how it can be leveraged to deliver evidence-based mental health interventions effectively.

**Objectives:**

The current study aimed to test the feasibility and acceptability of delivering this evidence-based intervention via social media, specifically Instagram. We hypothesized that the intervention would be both feasible (demonstrating high reach) and acceptable, generating better social media engagement metrics – such as higher link clicks and lower recruitment cost-per-click (CPC) rates, which indicate the intervention’s cost-effectiveness, compared to the control video.

**Methods:**

An eight-day Instagram campaign in February 2024 targeted U.S. youth aged 18-24. The campaign featured a 60-second personal story video, previously shown to reduce stigma among CT survivors, and a psycho-educational control video providing generic mental health facts, lacking social-contact elements. We assessed the campaign’s total cost-effectiveness and key social media engagement metrics: Impressions (number of times the video was displayed), reach (number of distinct viewers), link clicks (engagement with treatment resources), and recruitment cost-per-click (CPC).

**Results:**

The campaign generated 628,000 impressions, reached 209,000 Instagram users, and resulted in 4,015 link clicks, indicating its feasibility. The intervention video outperformed the control video in all measures, achieving 2,062 link clicks with a CPC of $0.79, compared to 1,953 link clicks and a CPC of $0.84 for the control group.

**Conclusions:**

The findings suggest that Instagram can effectively disseminate cost-effective interventions, potentially improving attitudes about CT and encouraging youth to seek help.

**Disclosure of Interest:**

None Declared

